# Art as a meaningful environment: an integrative model of aesthetic engagement and interpretation

**DOI:** 10.3389/fpsyg.2025.1677529

**Published:** 2026-01-21

**Authors:** Melissa J. Dolese

**Affiliations:** Department of Social Science, University of Alaska Southeast, Juneau, AK, United States

**Keywords:** proto-environment, art, aesthetics, embodiment, affordances, communication, distal, proximal

## Abstract

This paper proposes a novel framework for aesthetic experience by conceptualizing artworks as *proto-environments*—structured, dynamic spaces where meaning emerges through the synthesis of perception, cognition, and embodied interaction. This reframes art from a representational object to a bounded, communicative environment that actively invites engagement. While the model is developed through visual art, it generalizes across material, temporal, and conceptual forms. The framework rests on two integrated mechanisms. First, the distal–proximal continuum of aesthetic engagement differentiates modes of structural engagement along a fluid, non-categorical spectrum: distal engagement emphasizes imaginal projection, whereas proximal engagement involves direct sensorimotor interaction. Artworks typically activate both, producing a blended structural profile. Second, the functional-to-aesthetic stance serves as the interpretive filter that directs how the structural profile is understood. Drawing from the container schema, ecological affordance theory, and the cooperative principle, the stance determines whether embodied inputs are treated primarily as objective structure (external locus) or as non-utilitarian, communicative meaning (internal locus). Once the aesthetic stance is adopted, the artwork’s internal locus functions as a meaning-directed environment organized by the same fundamental dimensions as the physical world—space, time, and materiality. Aesthetic meaning emerges through the viewer’s embodied negotiation of this structured environment across the distal–proximal range. This perspective reframes artworks not as static messages to decode, but as dynamic fields of engagement whose aesthetic status is determined by how the stance prioritizes embodied inputs as metaphorical content, shaping how bodies move, feel, and think within both physical and imaginal space.

## 1 Art as a meaningful environment: introduction

Art has long been recognized as a form of communication, often analogized to language for its capacity to reflect human thought, emotion, and culture. Yet artworks are also environments. More than passive objects, artworks—whether material, conceptual, or temporal—are bounded spaces that can be projected into, inhabited, and unfold over time, influencing how we think, feel, and connect.

Artworks vary in the ways they prioritize the viewer’s body in the act of interpretation. Some invite imaginal entry, requiring projection into internalized spatial, temporal, and material dimensions; others demand bodily interaction, shaping perception through direct multisensory engagement, movement, and spatial negotiation. Understanding this spectrum of engagement provides a lens for analyzing how art functions as both a cognitive and affective environment.

The environmental dimension of art has been recognized as extending beyond the artwork to the space of its reception. Modern galleries, despite their apparent neutrality, function as powerful ideological containers that structure aesthetic experience, stripping artworks of original context and imposing a specific mode of contemplation (O’Doherty, 1999). Installation art extends this further by immersing viewers in experiences that dissolve boundaries between viewer and object, demanding embodied participation (Bishop, 2005).

Artists across the 20th and 21st centuries have increasingly explored forms of artistic practice that situate the viewer inside the work rather than before it (Bishop, 2005), foregrounding spatial design, bodily orientation, and multisensory experience as central components of meaning. This practice exemplifies how contemporary art leverages embodied navigation and spatial design to provoke sensory, affective, and cognitive responses. We see this in works ranging from digital reinterpretations of canonical pieces—like Van Gogh’s swirling night skies projected across walls, or Monet’s water lilies rippling across floors—to monumental installations such as Richard Serra’s tilting, curving steel sculptures, Yayoi Kusama’s mirrored *Infinity Rooms*, and James Turrell’s luminous chambers. These experiences highlight a crucial theoretical challenge: to model the dynamic and non-linear relationship between physical distance and embodied interpretation.

Van Gogh’s *The Starry Night* (1889) offers a clear example of how a single artwork can afford different modes of engagement depending on the medium through which it is encountered. In its original painted form, the work primarily invites distal-dominant engagement: the viewer stands outside the scene, mentally projecting into the swirling sky and simulating its movement and scale. In contrast, when the same visual material is translated into an immersive format—as in *Van Gogh Alive*—the mode of experience shifts. The work envelops the viewer, transforming distal imaginal inhabitation into a proximal, embodied form of spatial immersion.

The argument presented here introduces the neologism *proto-environment* to conceptualize distal-dominant artworks not as static representations or merely integrated within external sites, but as *living interfaces* that foreground how spatial and material structures actively solicit sensorimotor, affective, and interpretive engagement. The concept of the artwork as an internal cognitive and communicative container finds deep resonance in phenomenological accounts of Bachelard (1958, 2014) and formal grounding in the container schema—the fundamental cognitive structure through which we conceptualize objects as bounded entities with interiors and exteriors, derived from embodied cognition (Lakoff and Johnson, 1980).

Bachelard’s (1958, 2014) poetic and phenomenological perspective reveals how deeply enclosed spaces—from intimate nests to vast attics—carry immense psychological and imaginal weight. These are not merely physical structures; they stir and hold memory, emotion, and imagination, providing a bounded space for conceptualization. This resonance is often tied to an embodied meaning, in which the primal, non-cognitive experience of a space evokes physiological and emotional responses (Dolese and Kacinik, 2021). This embodied psychological resonance finds a clear parallel in Lakoff and Johnson’s (1980) container schema—an elemental conceptual structure that informs how we conceptualize emotions, relationships, identity, and meaning itself. The artwork’s boundedness guides interpretation by marking a perceptual “inside” where meaning resides. Artwork as a cognitive and communicative container—a metaphorical and ecological structure arises through which meaning is held, entered, and co-constructed. These containers structure engagement, including social and interactive affordances that shape relational as well as sensory experience.

Building on this understanding of artworks as bounded cognitive and communicative containers, proto-environments solicit engagement through both imaginal and embodied processes. This paper introduces the distal–proximal continuum of aesthetic engagement, a systematic framework used to describe and differentiate the blended structural profile of viewers’ cognitive and sensorial engagement with artworks along a continuum. At the distal end, engagement relies primarily on sensorimotor imagination (Freedberg and Gallese, 2007), allowing viewers to mentally inhabit space, simulate movement, and project affective states without interacting with the artwork in the concrete material space. Toward the proximal end, engagement increases through direct bodily interaction, as viewers explore and negotiate the artwork’s spatial and material affordances. Different senses and modes of interaction may be elicited across the artwork’s dimensions; however, the resulting experience is synthesized into a single structural blend, reflecting the relative contribution of distal and proximal processes.

Aesthetic meaning emerges from the blended structural profile of engagement, integrating imagination, perception, action, and embodiment. The interpretation of this emergent meaning is modulated by the functional-to-aesthetic stance. This stance governs the interpretive mechanism required for engagement, dictating the relative priority given to the internal locus (communicative and symbolic content—the “artified” dimension) versus the external locus (structural and material properties—the functional dimension). Because metaphorical interpretation only operates when the internal locus is prioritized, the degree to which an object is perceived as artified determines whether embodied inputs are treated as metaphorical content or as uninterpreted structure.

This framework draws on embodied cognition (Lakoff and Johnson, 1980; Wilson, 2002; Varela et al., 2017), ecological perception (Gibson, 1979), and linguistic pragmatics (Dolese and Kozbelt, 2021; Dolese et al., 2014; Grice, 1989, 1975). The structure of the paper builds this framework progressively. The *What* formalizes proto-environments by establishing their three core dimensions—space, time, and materiality—and elaborates the distal–proximal continuum and the functional-to-aesthetic stance. The *Why* argues that artworks evoke engagement by functioning as intentional, bounded, and communicative environments, detailing the pragmatic and cognitive mechanisms—consistent with Gricean principles—that elicit the aesthetic stance. The *How* specifies the mechanisms of aesthetic perception and embodied interpretation that are governed by the stance’s prioritization of the internal locus across the distal–proximal continuum of engagement.

Ultimately, this perspective reframes artworks not as static messages to be decoded, but as dynamic fields of engagement whose aesthetic status is determined by the functional-to-aesthetic stance’s prioritization of embodied inputs as metaphorical content, thus structuring how bodies move, feel, and think within and through both physical and imaginal space.

## 2 The what: structural dimensions of the proto-environment

The proto-environment is formally structured by the fundamental dimensions of the external physical world: space, time, and material/biophysical properties. These three dimensions are the organizational basis for aesthetic objects and experiences within this framework. Crucially, the observer utilizes metaphorical schemas to map non-spatial emotional and conceptual content onto these dimensions, facilitating embodied interpretation (Lakoff and Johnson, 1980) (see Table 1).

**Table 1:** Dimensions of the proto-environment model.

Dimension	Distal polarity	Proximal polarity
Space	Imaginal projection: mental construction of spatial relationships, scale, composition, or conceptual structure. In music or conceptual art, this includes pitch, tonal or thematic landscape, symbolic or relational “distance,” and metaphorical axes (e.g., tension–release, near–far in affective terms).	Proximal structuring: the artwork and its encompassing environment actively shape the body’s spatial relationship and movement—through architectural guidance, haptic proximity, physical navigation, or direct interaction with perceptible cues (e.g., stage placement, installation layout, movement through space).
Time	Imaginal flow: temporal structure is internally organized through attention, anticipation, and imagined sequence—how the viewer mentally constructs rhythm, pacing, narrative unfolding, or conceptual progression. Applies equally to music, performance, or sequential conceptual works.	Lived duration: time is experienced through direct bodily participation as the artwork unfolds—via movement, environmental change, or real-time interaction. Includes physical enactment in performance, engagement with durational installations, or attending to sequential sensory events.
Materiality	Sensorimotor simulation: the perceiver vicariously feels texture, weight, force, or symbolic presence through neural mirroring, embodied imagination, or interpretive inference. In abstract or conceptual works, “materiality” is experienced as intensity, density, or symbolic/semantic weight.	Multisensory registration: material presence engages the body through direct sensory input—touch, pressure, vibration, temperature, or resistance. In non-concrete media, this includes auditory, visual, or kinetic perceptual properties that are physically sensed.

While space, time, and material/biophysical properties are well-established concepts in aesthetic theory, their specific organization and functional role within this model are novel. The proto-environment framework synthesizes these three dimensions as the foundational organizing features of engagement, structuring how perceptual, imaginal, and embodied processes unfold during art experience. Crucially, it is the *pattern of engagement across these dimensions*—not the dimensions in isolation—that determines an artwork’s aggregate position on the distal–proximal continuum of embodied aesthetic engagement. This position is derived by assessing the degree to which processing is anchored in spatial, temporal, and material activity that is primarily external to the body (proximal-dominant) or internally simulated (distal-dominant), and by integrating these contributions into a single, unified continuum.

Space is engaged through the perceptual and relational structure of the artwork’s spatial environment. The brain computes scale, orientation, and boundaries that organize attention and movement within this cognitive–material space. Even in temporal or abstract works, the mind constructs an internal “spatial” framework, using sequences, phrasing, or implied movement to map relationships and trajectories within the work. These spatialized representations also support metaphorical mapping of non-spatial content—such as emotion, narrative progression, or affective tension—onto internal axes (e.g., UP–DOWN, PEAK–VALLEY), enabling the observer to navigate and inhabit the artwork’s structured environment.

Time is activated via rhythm, event boundaries, sequence, and duration, encompassing the flow of attention, narrative or musical sequence, and temporal flux of experience. This dimension is central to temporal arts such as music, literature, and performance, but is also subtly present in static visual forms through implied motion or repetition.

Material/biophysical properties invite either proximal engagement through physical interaction (e.g., walking through Richard Serra’s monumental steel sculptures to experience their scale, tilt, and spatial presence) or distal engagement via imaginal simulation (e.g., visualizing a rough texture in literature, painting, or implied motion in static works).

Collectively, these dimensions frame art as a structured context for meaning-making, organizing and guiding interpretation through embodied and situated engagement (Merleau-Ponty, 2012). Perception is not a passive reception of stimuli but emerges through sensorimotor negotiation with an environment’s spatial, temporal, material, and social affordances (Gibson, 1979; Reed, 1996; Fox and McEwan, 2017). Within the proto-environment model, these dimensions are situated along the distal–proximal continuum, which describes the relative blend of imaginal versus directly embodied processes recruited during engagement. The functional-to-aesthetic stance then modulates how this blended profile is interpreted by continuously weighting the internal and external loci, thereby influencing whether embodied inputs are treated as primarily structural/functional or as metaphorical and communicative. Together, the continuum and the stance provide an integrated account of how affordances shape both the mode and meaning of aesthetic experience.

### 2.1 Distal–proximal continuum of aesthetic engagement

The structural engagement between perceiver and artwork is defined by a single, unified distal–proximal continuum of aesthetic engagement. This continuum reflects the combined activation of sensory, imaginal, and embodied processes across the three core dimensions of space, time, and materiality. It is not dichotomous; artworks frequently recruit both distal and proximal processing simultaneously, generating a dynamic and blended profile. Distal engagement refers to processing through sensorimotor imagination and internal simulation (Freedberg and Gallese, 2007), constructing spatial, temporal, and material properties within imaginal space (e.g., narrative transportation, Green, 2004). Proximal engagement involves direct, multisensory interaction with the artwork’s material environment, where physical movement and tactile registration actively shape interpretation in real time. The continuum therefore captures a blended, dynamic profile of engagement, even as different dimensions contribute unequally at any given moment.

This spectral continuum is especially useful for complex or hybrid media. Temporal artworks such as music or sound installations engage the material/biophysical dimension proximally—sound waves are mechanically registered by the body—while the spatial dimension may be processed distally through metaphorical and affective schemas (e.g., ascent/descent, expansion/contraction) that structure an imaginal spatial environment. Artworks thus generate internal spatial fields that function as containers for emotional or conceptual movement even when no physical navigation occurs.

This combinatorial approach to perception is analogous to how the brain processes other sensory information. For example, both color (vision) and odor (olfaction) rely on combinatorial coding—the perception of millions of hues or thousands of distinct scents arises not from a one-to-one mapping of stimulus to unique receptor, but from interpreting the unique profile of activity across a limited population of receptors (e.g., three cone types for color, or ~400 olfactory receptors for scent). Similarly, the vast space of aesthetic possibilities is interpreted through the brain’s integration of the blended profile across the distal–proximal continuum and the three core dimensions of space, time, and materiality.

In sum, aesthetic experience is not captured by a single fixed point on the continuum, but by a dynamic, blended profile reflecting the synthesis of sensory, imaginal, and embodied engagement. This profile emerges from the specific locus of processing—distally, through sensorimotor simulation and imaginal construction, or proximally, through direct perception of material, spatial, and temporal properties—across the three dimensions. By accounting for this dimensional synthesis, the distal–proximal continuum provides a unifying lens for understanding how complex or hybrid media structure layers of activation and meaning.

### 2.2 The functional–to–aesthetic stance

The functional-to-aesthetic stance is the interpretive mechanism operating on the structural profile generated by the distal–proximal continuum. It is a graded, continuous orientation that establishes interpretive priority between the external locus and the internal locus. This stance governs the core decision that determines an object’s aesthetic status: whether embodied inputs are treated primarily as uninterpreted structure (external/functional loci) or as metaphorical and communicative content (internal/aesthetic loci). The distinct goals, processes, and consequences associated with each locus are summarized below (see Table 2).

**Table 2:** The functional–aesthetic stance: internal vs. external locus of interpretive priority.

Feature	Internal/communicative focus (aesthetic locus)	External/structural focus (functional locus)
Interpretive goal	Meaning inside the proto-environment: communicative intent, symbolic content, narrative, social or expressive meaning.	Structural reality: objective, measurable properties of the artwork (space, time, materiality) that constrain and shape interpretation.
Role of affordances	Affordances are interpreted as expressive cues, invitations for narrative or social engagement, or symbolic gestures (e.g., a path invites narrative exploration).	Affordances are registered as physical, spatial, or temporal features that enable action, navigation, or perceptual mapping (e.g., a path provides a navigable surface).
Primary cognitive process	Simulation, inference, and metaphor: Embodied cognition and neural mirroring enable vicarious experience and interpretation of “why.”	Sensorimotor registration and mapping: Perception and action systems enable understanding of “what” and “where.”
Stance function	Priority moves toward the internal locus, increasing the weighting of communicative meaning in the blended profile of engagement.	Provides the baseline structure of the object that can be interpreted, influencing the blended profile but not defining meaning by itself.
Example	Interpreting a photograph’s composition as conveying social power dynamics or narrative intent.	Registering the precise volume, tempo, and pitch variation of a musical piece; or registering the physical dimensions, surface texture, and light reflection of a canvas.

Most engagement involves simultaneous recruitment of both loci. The functional–aesthetic stance modulates their relative contribution, moving the profile along a graded continuum rather than switching categorically between poles. The same affordances or cognitive processes can support both distal/imaginal and proximal/sensorimotor engagement depending on context, viewer skill, and interpretive orientation.

Aesthetic meaning emerges from the blended structural profile of engagement along the distal–proximal continuum. The continuum defines the mode of engagement, capturing how imagination and embodiment are integrated, while the functional-to-aesthetic stance determines interpretive priority, framing that engagement as communicative or functional. The stance dictates whether the blended profile is interpreted primarily through the internal locus (metaphorical content) or the external locus (uninterpreted structure) without altering the structural continuum itself. This integrated framework draws on embodied cognition (Lakoff and Johnson, 1980; Varela et al., 2017), ecological perception (Gibson, 1979), and linguistic pragmatics (Dolese et al., 2014; Grice, 1989, 1975).

## 3 The why: art as a communicative environment

Humans are cultural beings who seek connection, communicate through language, and perform ritual and creative acts—a proclivity that underpins the deeply social role of art in human development (Dissanayake, 1992, 2015b; Tomasello, 2009). Art functions as a fundamentally social and communicative space, designed not merely to contain meaning but through its affordances to invite exploration, interpretation, and the co-construction of that meaning (Dolese et al., 2014; Tomasello, 2009). This social grounding is exemplified by temporal arts such as song and dance, theorized to have emerged from adaptive behaviors like mother–infant bonding and group cohesion (Dissanayake, 2015b; Greenberg et al., 2021). These ritualized behaviors evolved to regulate emotion and strengthen social bonds, with neurochemical rewards—oxytocin and dopamine—released during communal art activities (e.g., music-making, dance), providing physiological reinforcement for art’s vital social function (Greenberg et al., 2021; Lacey et al., 2011).

This social grounding is further supported by the extended self hypothesis (Belk, 1988), which implies that the artwork can be treated as an intentional extension of the artist. Engagement with an artwork thus constitutes a social interaction in which meaning-making is inferred from projected communicative intent. Just as humans possess internal and external spaces, the artwork is implicitly granted both a phenomenal interior (containing abstract concepts and intentions) and an external material form. In this framework, the internal environment locus functions as the container for metaphorical and communicative meaning, structured along the dimensions of space, time, and materiality, while the external environment locus represents the artwork’s tangible, physical manifestation. These loci govern how the blended profile of engagement—whether distal or proximal along the distal–proximal continuum—is interpreted, reinforcing the artwork’s status as a proto-environment where both imagined and embodied interactions are meaningfully integrated.

### 3.1 The proto-environment as communicative container

This communicative challenge is resolved by the artwork operating as a proto-environment—a bounded, perceptually and affectively structured space where meaning is actively encountered. Art, while not a language in a strict sense, communicates through its inherent properties: materiality (material/biophysical), form and spatial interaction (space), and composition and rhythm (time). The container schema (Lakoff and Johnson, 1980) provides a cross-modal cognitive structure in which experiences are conceptualized as bounded spaces that contain, organize, and constrain elements. Applied to art, this schema allows viewers to mentally “enter” the artwork, integrating sensory, spatial, temporal, and conceptual information into a coherent environment that can be explored both perceptually and imaginatively. This communicative potential manifests through social affordances: perceived opportunities for connection, communication, and shared understanding (Fox and McEwan, 2017; Reed, 1996). Even when physical affordances are minimal, these social affordances prompt viewers to treat the proto-environment as a site of dialogue, elaborated by the art-as-communication framework (Dolese et al., 2014).

Building on this cognitive foundation, Gibson’s (1979) ecological approach explains active engagement with these meaning-laden environments. Perception is not passive reception of sensory data but an active pickup of information from the environment through affordances—the action possibilities that objects, surfaces, and events offer based on individual capabilities. The artwork environment is not neutral. Artworks are structured environments offering affordances—possibilities for action and interpretation—to perceivers, which vary depending on viewer position, attention, experience, and movement.

For example, two-dimensional paintings offer physical affordances—flatness, surface, weight—that often mismatch their communicative purpose. A canvas could physically function as a tray table or window cover, but these uses are subordinate to its primary design. Its material affordances do not align with practical function, signaling primary affordances as communicative, not instrumental—thus prompting the aesthetic stance. The artwork’s physical structure invites interpretive action: looking, feeling, thinking, and connecting. The art object makes its purpose visible as a social affordance: a space of exchange between artist and viewer, where meaning, emotion, and communicative intent are negotiated.

This interpretive flexibility, in which an object’s affordances can be read as functional, aesthetic, or a combination of both, reflects a longstanding tension in aesthetics between form and function (e.g., Bell, 1916; Sibley, 2001). In this framework, that tension is formalized through the functional-to-aesthetic stance, which integrates Gibson’s (1979) ecological theory of affordances, Dissanayake’s (1980) and Dissanayake (2015a) theory of artification, and Gricean pragmatics (Grice, 1975). When an object’s perceived purpose is predominantly utilitarian, observers adopt a functional stance, prioritizing physical and instrumental affordances such as durability, stability, or efficiency. Deviations from utilitarian expectations—contextual and culturally mediated cues such as ornamentation, fragility, or exaggerated scale—shift interpretive priority toward the aesthetic stance, emphasizing communicative intent, relational meaning, and cognitive affordances. Importantly, the distal–proximal continuum describes the mode of engagement (the relative contribution of imaginal versus directly embodied processes), whereas the functional-to-aesthetic stance determines interpretive priority along the internal and external loci. The proto-environment itself always exists, but the degree to which an observer perceives and interprets it aesthetically versus functionally depends on the graded, continuous operation of the stance. A single object can evoke predominantly functional engagement, predominantly aesthetic engagement, or a nuanced blend of both. The aesthetic stance becomes dominant when communicative intent is pragmatically inferred as central, consistent with Gricean principles of relevance (Dolese and Kozbelt, 2021; Dolese et al., 2014).

### 3.2 Pragmatic inference: the Gricean framework

With the cognitive framework (container schema) and interactive possibilities (affordances) established, cooperative principle of Grice (1975) and Grice (1989) illuminates how meaning is organized and interpreted in these exchanges. Participants implicitly adhere to a principle requiring contributions to align with the accepted purpose of the interaction. This principle is broken down into four maxims: quality (truthfulness), quantity (informativeness), relation (relevance), and manner (clarity).

Though developed for linguistic conversation, this framework can be meaningfully applied to visual artworks, treated as intentional communicative gestures. Aesthetic appreciation is influenced by perceived adherence to, or violation of, these maxims. For example, quality relates to truthful representation or material integrity; quantity to visual information density; relation to cultural or personal relevance; and manner to clarity or coherence of form. Empirical work shows endorsement of these principles correlates with aesthetic liking and expertise. Artists tend to interpret abstract works as intentionally communicative, whereas non-artists may perceive ambiguity as a communicative breakdown rather than deliberate choice. Violations often function generatively, producing tension, irony, or resonance that demands inferential labor from viewers, thereby eliciting the aesthetic stance and prioritizing the Internal Locus for the negotiation of metaphorical content.

### 3.3 Functional-to-aesthetic shift case study: Duchamp’s *bicycle wheel*

This framework helps illuminate how social affordances operate in art, especially in cases where physical affordances are diminished or subverted. A prime example is Marcel Duchamp’s 1913 readymade—*Bicycle Wheel*, which consists of a bicycle fork and wheel mounted upside-down on a wooden stool. By rendering both components non-functional, Duchamp deliberately strips them of their expected physical affordances (the stool no longer affords sitting, the wheel cannot rotate for transportation). This stripping of utility forces a functional-to-aesthetic shift. In removing the utility, Duchamp exposes the object’s communicative potential. The work thus becomes a symbolic gesture—a commentary on the nature of art, intention, and interpretation, highlighting how conceptual art is primarily driven by ideas rather than material manipulation (Goldie and Schellekens, 2007). In terms of communication theory, this represents a deliberate violation of the quality maxim (the rule of representational truthfulness), while simultaneously adhering to the relation maxim, as the piece remains highly relevant within the discourse of modern art.

Notably, the violation of physical affordances acts as a primary sensory cue signaling communicative intent. This sequence prompts viewers to adopt the functional-to-aesthetic stance and prioritize the internal locus, locating metaphorical meaning and resolving the apparent contradiction of the object’s non-functionality.

### 3.4 Inference, embodiment, and neural correlates

Social affordances operate through inference and projection. Meaning and communicative intent are inferred in interpersonal interactions via gesture, tone, and facial expression (Preissler and Bloom, 2008). Similarly, engaging with a painting involves deciphering the artist’s communicative intent through composition, symbolism, and material choices. These cues invite projective entry into a proto-environment, where meaning is co-created rather than dictated. Ambiguity is not a flaw but a precondition for imaginal projection. Jakesch and Leder (2009) found viewers prefer moderate ambiguity in artworks. Images too explicit or obscure limit personal projection, whereas ambiguous art opens a conceptual space—a mental proto-environment—where individual experiences and interpretations are projected. Neurocognitive research further reveals the body responds similarly to imagined and real stimuli (Jeannerod, 2001; Gallese, 2007), reinforcing that mentally or emotionally “stepping into” an artwork engages embodied systems across the distal–proximal continuum. As an inherent aspect of human culture, art is often expected to reflect shared experiences and be broadly comprehensible, especially in public or institutional contexts. However, many artworks—particularly abstract or conceptual—function as distal proto-environments, requiring cultural knowledge, imagination, or emotional openness to enter. Lack of interpretive tools, such as familiarity with artistic styles or conceptual frameworks, may lead to alienation or exclusion (e.g., Landau et al., 2006; Snapper et al., 2015). This interpretive gap highlights the importance of framing artworks not as static objects but as environments affording varying degrees of embodied and cognitive entry.

Neural correlates of art reception further support the social communicative role of art. At the neural level, the reception of art also activates reward and empathy circuits, reinforcing the biologically rewarding nature of aesthetic engagement (e.g., Lacey et al., 2011). Comprehensive reviews of the neural correlates of visual aesthetic appeal highlight the involvement of various large-scale brain systems, including higher-level visual processing, subcortical reward pathways (such as the ventral striatum), and particularly the prefrontal cortex and default mode network (DMN), emphasizing that aesthetic appeal arises from the complex interaction between a stimulus and the observer (Vessel et al., 2022; e.g., Chatterjee and Vartanian, 2014). fMRI studies indicate that when viewers believe a painting is human-made rather than AI-generated, activity increases in brain regions linked to self-referential thought, narrative understanding, and social cognition—especially the medial orbitofrontal cortex and DMN (Kirk et al., 2009). These findings suggest that even without physical interaction, art functions as a social signal inviting projection, empathy, and cognitive resonance.

Art thus functions as a dynamic communicative environment, inviting active engagement and the co-creation of meaning. Artworks emerge not as static carriers of content but as relational fields—structured yet permeable—where cognition, perception, and culture converge. In navigating, inhabiting, and projecting into the artwork’s proto-environment, viewers experience art as both a personal and social space, a context in which interpretive, affective, and embodied processes unfold simultaneously. This relational and experiential perspective sets the stage for the How, which examines the specific mechanisms by which art structures perception and guides embodied interpretation across the distal–proximal continuum.

## 4 The how: mechanisms of aesthetic perception and embodied interpretation

Having established why artworks function as profoundly meaningful proto-environments through their social affordances and communicative structures, the focus now shifts to how the structure of these environments enables interpretation. The artwork defines a single, cohesive internal/external environment interface that serves as the locus of its interdependent dimensions: a phenomenal interior (the internal environment) where meaning and concepts reside, and a perceptible structure (the external environment) that is sensory-manifested. The distal–proximal continuum (introduced earlier) maps this integrated relationship, describing the blendable spectrum of engagement that ranges from emphasizing the artwork’s abstract, imaginal interior (distal polarity) to directly engaging its sensory-manifested structure (proximal polarity). This continuum is crucial because the artwork’s internal environment (the container for abstract meaning) is universally conceptually structured by metaphorical space, time, and materiality, allowing the viewer to map conceptual content and meaning using the same embodied frameworks that organize the artwork’s sensory manifestation.

This engagement is not uniform. While the initial adoption of the aesthetic stance relies on universal perceptual signals, the subsequent process of co-constructing meaning—particularly with abstract or conceptual art—often requires specific cultural knowledge, imaginal capacity, or emotional openness for full immersion. When viewers lack the necessary interpretive tools (e.g., familiarity with artistic styles or conceptual frameworks), they may experience alienation or exclusion, as they lack the scaffolding needed to map meaning onto ambiguous representations (Dolese et al., 2014; Landau et al., 2006). This interpretive gap underscores the need to analyze the precise “how” of art engagement: the specific mechanisms by which artworks afford varying degrees of embodied and cognitive engagement, and how these mechanisms interact with both universal human perceptual systems and diverse, acquired cultural frameworks. This section explores these processes, varying along the distal-proximal continuum, to clarify the dynamic interplay between fundamental human capacities and acquired cultural knowledge in shaping aesthetic experience.

### 4.1 Universal principles of aesthetic structure and perception

Viewers’ active engagement with proto-environments begins with the perception of fundamental compositional elements like line, form, shape, directionality, and balance, which combine to create artistic objects. From the moment of first perception, meaning is disambiguated through embodied processes facilitated by a dynamic brain–body–environment feedback loop (Bilda et al., 2007; Bruineberg, 2018).

Principles of aesthetic perception further reveal how artists harness compositional elements to evoke responses. Drawing from Fechner’s foundational work (Norman et al., 2010) and Berlyne’s arousal theory (Berlyne, 1960, 1970; Yerkes and Dodson, 1908), studies show how elements like complexity (Eisenman, 1966) and preference for curved over angular lines (Cotter et al., 2017; Gómez-Puerto et al., 2016, 2018; Palumbo et al., 2015) shape initial visual experience. These preferences likely stem from embodied encounters with smooth, safe natural forms.

Beyond the impact of individual elements, the arrangement of these forms also profoundly influences aesthetic judgment. The concept of “visually right” design—intuitive or learned arrangements that feel balanced and pleasing—has been empirically supported through studies showing that balance functions as a key organizing principle in visual composition (Locher et al., 1998). Research on Mondrian’s abstract paintings further demonstrates viewers’ sensitivity to orientation and compositional structure (Hekkert and van Wieringen, 1996; Mather, 2012; Leeuwenberg and Van der Helm, 2013), showing that deviations from these principles disrupt perceptual fluency and aesthetic preference. This fluent processing fosters a coherent and engaging visual environment.

Research by Arnheim (1954) shows that perception is inherently shaped by organizing tendencies of the mind, and artworks capitalizing on visual forces like balance and tension engage the viewer’s perceptual systems. Similarly, Hekkert (2006) argues aesthetic pleasure in design emerges from principles like unity-in-variety and fluency, while Locher (2006) demonstrates how compositional structure guides eye movements and contributes to meaningful aesthetic appraisal. These perspectives support that compositional and formal aspects of visual art are not arbitrary but contribute to meaning-making. Norman et al. (2010) further argue that aesthetic judgment emerges from a dynamic interplay between bottom-up perceptual cues and top-down cognitive processes. Together, these accounts suggest that perceptual structure scaffolds embodied aesthetic engagement, with the final integration of sensory, affective, and cognitive dimensions emerging through the viewer’s dynamic engagement rather than conscious interpretation.

Color, as a fundamental compositional element, also plays a significant role in shaping an artwork’s cognitive and emotional landscape. Cross-cultural research on color-emotion congruence suggests that these associations are not arbitrary (Jonauskaite et al., 2020). Some theories propose that color meanings are grounded in average affective responses to objects typically associated with those hues in our environment (Palmer and Schloss, 2010). Such learned associations contribute to the emotional tone derived from the visual scene. For example, culturally or personally prevalent colors can evoke strong emotional reactions within an artwork, shaping both cognitive and embodied engagement with its atmosphere.

### 4.2 Distal engagement: projective immersion in two-dimensional proto-environments

Having established the universal principles by which artworks are structured, attention now turns to how these principles specifically manifest and are engaged within distal proto-environments. In such environments—like two-dimensional paintings—meaning-making relies heavily on the viewer’s ability to mentally project into the artwork’s designed space and engage in embodied simulation (Freedberg and Gallese, 2007), also termed sensorimotor imagination. Even what appears to be a passive activity, like the visual interpretation of a flat painting, activates complex sensorimotor processes and imaginal simulations (e.g., Jeannerod, 2001; Varela et al., 2017). This process involves perceptual input triggering internal bodily states and action possibilities without overt physical movement. The interpretive mode requires the viewer to draw upon embodied experiences and cognitive frameworks to complete the meaning offered by the artwork (Arnheim, 1954; Hekkert and Leder, 2008).

Our initial engagement with two-dimensional works, especially paintings, involves processing them holistically, similar to the rapid extraction of “gist” that occurs when viewing real-world scenes. Despite being flat and visually distal, such 2D artworks are reliably processed as unified, structured environments, with participants discerning an image’s gist even when flashed briefly (Greene and Oliva, 2009a, 2009b). This rapid extraction is mediated by the visual system processing physical and semantic regularities—global features such as naturalness, openness, roughness, expansiveness, and color (Shottenkirk et al., 2019). These global features are precisely the organizational structures that define a proto-environment, mapping onto the core dimensions of metaphorical space (openness, expansiveness), materiality (roughness, color), and implied time (narrative sequence or flux). By rapidly grasping the essence and meaning of these environmental features, the viewer is prepared for imaginal projection into the artwork’s conceptual space (Greene and Oliva, 2009a, 2009b).

Another vital pathway for embodied interpretation—particularly salient in distal engagement—involves the mirror neuron system (Gallese and Freedberg, 2007). These specialized neurons activate both when an action is performed and when others are observed performing it, enabling internal simulation of movement, effort, and intention. This embodied simulation applies to both the artist’s creation and the viewer’s perception, as neuroaesthetic research highlights (Chatterjee and Vartanian, 2014; Gallese and Freedberg, 2007). For example, observing a bold, gestural brushstroke may evoke an internal sensation of the sweeping motion, conveying the artist’s energy and dynamism. Conversely, witnessing delicate, layered paint can trigger sensorimotor simulations reflecting care and precision. This vicarious experience, facilitated by mirror neurons, exemplifies *enactive embodiment*—a mode of cognition where perception arises through simulated bodily states rather than passive observation. Viewers internally rehearse gesture, force, and affect, deepening sensorimotor resonance with the artwork.

Beyond individual compositional elements and direct embodied simulation, the artwork’s broader impact on cognitive and emotional circuits is also crucial. Neurally, art reception engages reward and empathy circuits, reinforcing aesthetic engagement’s biologically rewarding nature (Chatterjee and Vartanian, 2014; Leder et al., 2004; Lacey et al., 2011). For instance, an fMRI study revealed increased activity in regions linked to self-referential thought, narrative understanding, and social cognition—especially the medial orbitofrontal cortex and default mode network—when participants believed a painting was human-made rather than AI-generated (Kirk et al., 2009). This suggests that even without physical interaction, viewers respond to art as a social signal inviting projection, empathy, and cognitive resonance.

Ultimately, this multifaceted initial embodied interpretation—encompassing scene perception, mirror neuron simulation, and the influence of aesthetic principles (including balance, composition, and color)—lays the groundwork for the engagement of social affordances and more complex cognitive processing in the overall meaning-making experience.

### 4.3 Proximal immersion: the embodied turn in three dimensions

Two-dimensional artworks, functioning as distal proto-environments, tend to emphasize internal sensory-motor systems through imaginal projection. Moving into three dimensions marks a fundamental re-prioritization toward direct, sensorimotor interaction with artistic environments. This re-orientation—from a distal focus on imaginal entry to a proximal focus on physical immersion—significantly expands possibilities for embodied meaning-making (Varela et al., 2017).

Two-dimensional works foreground imaginal “entry,” functioning as proto-environments that engage scene processing and embodied simulation for vicarious experience (e.g., paintings, drawings, photographs, street art). By contrast, three-dimensional forms overtly invite spatial and tactile interaction, shifting the primary processing load to the proximal pole (Varela et al., 2017). This direct engagement requires sensorimotor coupling with the perceptible structure. Importantly, these proximal environments still maintain the Internal Locus, as their meaning—interpreted as the communicative or symbolic content “inside the container”—is negotiated through the body’s interaction with the artwork. In this context, the art object and the space it occupies form a unified proto-environment, extending communicative intent by structuring the surrounding space and transforming passive surroundings into an active field for embodied interaction.

These three-dimensional forms include sculpture, fashion, ceramics, and architecture (Noë, 2004; Malafouris, 2013). For instance, sculpture reshapes its surroundings, offering multiple vantage points and haptic qualities enriched by movement and proximity. Circling a sculpture or observing shifting light on textured surfaces fosters embodied understanding through real-time, situated perception, aligning with ecological cognition perspectives that emphasize sensorimotor coupling between agent and environment.

Fashion further exemplifies embodied engagement by stimulating exteroceptive (color, form), tactile (texture), and proprioceptive (movement) systems. As the body navigates fabric, cut, and motion, meaning is co-produced via dynamic feedback between material and a body in motion. Physical affordances—garment cut, weight, elasticity—shape interaction, creating an artistic experience worn rather than merely viewed (Entwistle, 2000; Bilda et al., 2007).

Architecture deepens sensorimotor participation, enlisting brain spatial navigation systems like the parahippocampal place area and grid cells (Moser et al., 2008). Environments are perceived not only visually but also through bodily orientation, temperature, echo, and ease of passage. Designers modulate sensory engagement through spatial transitions—as exemplified by Disney Imagineering and experiential designs such as mazes and labyrinths, which influence arousal and affective states by guiding sensory input and bodily movement. Mazes induce arousal and disorientation by limiting sensory input and choice, while labyrinths guide the body gently, producing calming effects through rhythmic bodily engagement (Behman et al., 2018; Sternberg, 2009).

Beyond large-scale environments, design objects such as musical instruments, mugs, and vases also communicate affordances through physical features. A violin’s neck directs hand positioning just as a vase’s opening suggests flower arrangement. These objects are both functional and communicative, eliciting touch, posture, and interaction—microcosms of embodied environments (Bilda et al., 2007; Krippendorff, 2005; Norman, 2007).

Contemporary immersive experiences push proximal engagement even further. Virtual reality (VR), augmented reality (AR), interactive installations, and participatory performances create fully realized proto-environments that blur the lines between artwork and lived space. VR envelops viewers in sensorily rich artificial worlds, engaging vision, audition, and sometimes haptics. AR layers digital content onto physical environments, creating a layered sensorium that modulates perception. Participatory art makes viewers co-creators, completing works through action. These formats maximize multisensory input and demand physical presence, generating meaning not through observation alone but through embodied negotiation (Bailenson, 2018; Bilda et al., 2007; Loomis et al., 1999; Varela et al., 2017).

Across this continuum—from distal to proximal art environments—the territory of aesthetic experience expands. As artistic forms increasingly enlist the full sensorium, they create spaces not only seen but inhabited. This embodied turn marks a fundamental shift from artwork as representation to an environment of interaction—a site where cognition is enacted, meaning co-constructed, and the body is a necessary agent of understanding (Varela et al., 2017).

## 5 The continuum of aesthetic experience: conclusion

Artworks are reconceptualized as proto-environments—dynamic, bounded spaces where meaning is perceptually, cognitively, and affectively constructed through active viewer engagement. This conceptualization is grounded in the fundamental human drive for *artification* (Dissanayake, 1980, 2015a), a universal behavioral tendency to make objects and experiences meaningful and socially significant through deliberate aesthetic elaboration.

The history of art reflects a continuous conceptual and material exploration of the relationship between the artwork and the embodied viewer—a theme philosophically and critically articulated by phenomenologists (e.g., Merleau-Ponty, Bachelard) and art theorists and historians (e.g., O’Doherty, Bishop). While distal proto-environments (such as painting) continue to solicit imaginal projection and internal simulation, art practice has long challenged the boundaries of the body through proximal forms such as installation, performance, and monumental sculpture. The most recent and significant expansion along the distal–proximal continuum is driven by immersive technologies (e.g., VR/AR). This trajectory validates the growing focus on embodied cognition in aesthetic theory, leveraging the artwork’s design to shift engagement from primarily cognitive interpretation to direct sensorimotor coupling. This reframing redefines art from a representational object to an environment of interaction, where cognition is enacted and the body becomes central to understanding.

The proto-environment model synthesizes three complementary theoretical perspectives that justify art’s role as a structured, communicative system. The container schema (Lakoff and Johnson, 1980) provides a cognitive template for perceiving bounded spaces of meaning. Ecological affordance theory (Gibson) explains how artworks actively invite engagement through perceived opportunities for action, including social affordances. Finally, Inferential Pragmatics (Grice) illuminates how meaning is not merely extracted but co-created through the violation of communicative norms. Ultimately, meaning emerges through a dynamic coupling of brain, body, and environment—a hallmark of the embodied mind framework.

### 5.1 Synthesis: the two worlds of aesthetic experience

The distal–proximal continuum specifies the structural mechanism of aesthetic engagement, describing how embodied and imaginal processes combine to form an integrated profile shaped by an artwork’s spatial, temporal, and material organization. This continuum captures the mode of engagement—the relative blend of distal (imaginal, internally simulated) and proximal (direct, sensorimotor) processing—but operates independently of any cultural, symbolic, or communicative interpretation the artwork may elicit. In most artworks, distal and proximal processes coexist to varying degrees, producing a dynamic and graded profile rather than a categorical distinction.

Aesthetic experience becomes fully realized when this structural “how” is joined with the functional–aesthetic stance, a graded orientation that governs the why of meaning—how observers infer purpose, significance, and communicative intent. The Stance modulates the interpretive balance between the internal locus (meaning generated within the artwork’s constructed proto-environment) and the external locus (the constraints and affordances imposed by the work’s material, spatial, and temporal structure). By clarifying the independence and complementarity of these two systems—the structural continuum of engagement and the evaluative stance that orients interpretation—the proto-environment model provides a unified, empirically tractable framework for studying aesthetic experience across media. It positions the embodied mind as the necessary integrator of perceptual, cognitive, affective, and social processes, explaining how artworks become experientially and meaningfully alive for observers.

## 6 Future directions: operationalizing the distal–proximal continuum of aesthetic engagement

The proto-environment framework offers a synthesis across aesthetics, cognitive science, perception, and communication. By reconceptualizing artworks as bounded environments for embodied interaction, it provides a roadmap for investigating how meaning emerges across diverse modes of engagement. The framework distinguishes between the structural mechanics of engagement, captured by the distal–proximal continuum, and the interpretive weighting of meaning, governed by the functional–aesthetic stance.

Structural measurement (continuum): The distal–proximal continuum captures how embodied and imaginal processes integrate across space, time, and materiality, producing a blended profile of engagement. This profile is continuous, reflecting the relative contributions of distal (imaginal, simulated) and proximal (direct, sensorimotor) processing.
Interpretive modulation (stance): The functional–aesthetic stance is a graded orientation that continuously modulates attention between the external locus (structural properties of the artwork) and the internal locus (communicative and symbolic meaning). It determines how observers interpret the blended structural profile, highlighting the meaning that emerges from engagement rather than imposing a categorical designation of “art.”

### 6.1 Functional-to-aesthetic stance: internal vs. external locus

The functional-to-aesthetic stance operates on the structural inputs provided by the distal–proximal continuum, determining how these inputs are interpreted. When the internal locus (aesthetic pole) is prioritized, engagement is directed toward communicative, metaphorical, or symbolic meaning within the proto-environment. When the external locus (functional pole) is prioritized, attention focuses on the artwork’s material, spatial, or temporal structure for functional purposes. Operationalization can include behavioral, self-report, or neural markers. Internal locus indicators (I) may include inferential reasoning about intention, engagement of theory of mind or social cognition networks, or tasks that probe symbolic interpretation, while external locus indicators (e) may include attention to measurable structure, motor readiness, or direct perception of physical affordances.

A stance score can be calculated as the relative weighting of internal versus external locus indicators:

Stance score = I/(I + E).

A score approaching 1 reflects strong aesthetic/internal prioritization, whereas a score approaching 0 reflects strong functional/external prioritization. This measure provides a quantitative handle on how the distal–proximal structural profile is interpreted along the functional–aesthetic spectrum, linking embodied engagement with the viewer’s evaluative orientation.

### 6.2 Operationalizing the continuous scale

The distal–proximal continuum captures how viewers engage with an artwork’s structure along a fluid spectrum. Distal engagement emphasizes sensorimotor imagination and internal simulation, whereas proximal engagement involves direct, multisensory interaction. To operationalize this continuum, researchers can derive a blended profile score (BPS) by quantifying the relative contributions of distal (D) versus proximal (P) processes across the core dimensions of space, time, and materiality. Importantly, distal and proximal processes often interact: distal simulations may rely on proximal sensory input, while proximal engagement can trigger imaginal projection. The BPS thus reflects the *dominant mode of engagement* rather than implying strict independence of these processes.

#### 6.2.1 Distal (D) markers

Quantify imaginal and simulated processes, such as activation of scene construction or neural mirroring networks, inferential reasoning scores, or self-reported internally generated mental imagery. These processes support the construction of internal, vicarious experiences of the artwork’s spatial, temporal, and material properties. When the object is perceived as artified—shaped by cultural conventions, contextual cues, or individual expectations (internal locus)—these simulations facilitate metaphorical and communicative interpretation.

*Primary cognitive process:* Simulation, inference, and potential metaphorical mapping contingent on artification.

#### 6.2.2 Proximal (P) markers

Quantify direct sensorimotor registration, including precise perception of haptic, auditory, or visual input, quantified motor preparation and interaction with the artwork, or eye-tracking metrics focused on structural features. These markers represent the body’s immediate, embodied perception of the artwork’s material, spatial, and temporal properties.

*Primary cognitive process:* Sensorimotor registration and embodied mapping.

The BPS is calculated as the ratio of these combined process scores:


$$\begin{array}{l} \mathrm{BPS}=(D\_\text{Space}+D\_\text{Time}+D\_\text{Materiality})/\\ (P\_\text{Space}+P\_\text{Time}+P\_\text{Materiality}). \end{array}$$

Higher BPS values (D/P ratio > 1) indicate relatively stronger distal, imaginal processing, while lower values (D/P ratio < 1) indicate relatively stronger proximal, direct engagement. Calculating the BPS requires integrating multiple modalities—behavioral, neuroimaging, and self-report data—to capture the full spectrum of engagement. In this way, the BPS provides a quantitative handle on the relative weighting of distal and proximal processes, linking the structural mechanics of embodied engagement with the interpretive orientation of the viewer.

### 6.3 Comparative designs across the spectrum

Empirical designs can compare the same artistic content in distal versus proximal forms, e.g., two-dimensional versus immersive VR installations. These comparisons track how cognitive, affective, and sensorimotor signatures shift along the distal–proximal continuum, revealing the dynamic interplay between structural engagement and interpretive weighting.

## 7 Conclusion

The proto-environment framework offers an embodied, ecological account of art, integrating the distal–proximal structural continuum with the graded modulation of meaning by the functional–aesthetic stance. This perspective positions the observer as the integrator of perceptual, cognitive, and affective processes, framing artworks as dynamic proto-environments whose communicative and aesthetic significance emerges through situated engagement. The model provides a foundation for empirical investigation of aesthetic experience across media, cultures, and immersive contexts.

## Data Availability

The original contributions presented in the study are included in the article/supplementary material, further inquiries can be directed to the corresponding author.
